# Quarantine

**Published:** 1897-03-20

**Authors:** 


### Medical Progress and Hospital Clinics.

[*The Editor will be glad to receive offers of co-operation and contributions from members of the profession. All letters should be addressed to* The Editor, at the Office, 28 & 29, Southampton Street, Strand, London, W.C.]

### QUARANTINE.

The Milroy Lectures, Delivered at the Royal College of Physicians, on March 11th, 16th, and 18th, by William Collingridge, M.A., LL.M., M.D., D.P.H. Cantab, Medical Officer of Health of the Port of London.

The lecturer said that the laws of quarantine, whatever form they might take, were framed with the idea of protecting a nation from the importation of disease, a risk which was practically in proportion to the extent of its trade. Few subjects, then, could have the same vital interest for a country such as England, for on the one hand was the extreme importance of free and untrammelled commerce, and on the other the no less essential point of safeguarding in every way the public health. After a careful history of the subject from the earliest times, it was stated that in 1873, so far as cholera was concerned, quarantine was officially abandoned by England, and medical inspection substituted for it, by an order of the Local Government Board. In 1874 an international congress was held at Vienna with the object of bringing about uniformity of action against imported disease, and a resolution was passed to the effect that it was necessary to establish in the ports of Europe, instead of quarantine, a rigorous sanitary inspection of every vessel coming from an infected port, and thus for the first time was medical inspection formally adopted in the place of quarantine by a majority of the European Powers.

In 1883 regulations issued by the Local Government Board gave power to the medical officer to visit and examine any vessel arriving from an infected port, and if he believed the vessel was "infected" to keep her entirely under his control. In 1890 power was given to the medical officer of health to require, in the case of an infected vessel, the name and address of all passengers who were found on medical inspection to be healthy before they were allowed to leave; and, by forwarding the names to the sanitary authorities of the districts in question, it was left in the power of each district to protect itself by keeping such persons under observation so long as might be deemed desirable. At last, in 1896, the Public Health Act became law, by which the powers of the Privy Council to deal with cholera, yellow fever, and plague were transferred to the Local Government Board, and quarantine in England was formally abolished; the protection of England against the importation of infectious disease lying for the future in medical inspection; a healthy vessel being no longer detained merely because she had arrived from an infected port.

The lecturer then spoke of the chief causes which had led to the abandonment of quarantine; among which were, that it had never been found possible to maintain it absolutely, so that a country had been subjected to a barbarous and costly system without even the satisfaction of knowing that the experiment was complete; that where carried out as strictly as possible it had not had the effect of arresting the importation of disease; and that sanitation and medical inspection had proved more efficacious and less burdensome. It was scarcely possible for those not actually interested in shipping to realise how serious a matter quarantine had been for shipowners, not only in the remote past, but even up to a very recent date, sometimes owing to the delay and uncertainty of detention, sometimes to actual prohibition of intercourse with ports supposed to be infected. So recently as 1827 all vessels bound up the river Thames, from ports east of Gibraltar, were compelled to run into the Medway and perform quarantine in Stangate. Such a waste of time and money would be utterly out of the question at the present day.

A very strong argument against quarantine was derived from the fact that in consequence of its being so often imposed on the merest suspicion every effort was made to hide the existence of epidemic disease. This policy of concealment had been constantly adopted in order to avoid the interference with trade which might follow the declaration of the presence of an epidemic, and this desire to keep the true facts secret prevented the authorities from taking proper measures to control the spread of infection. Many illustrations were given of the costliness of quarantine, as well as of its uselessness, and it was pointed out that not only was quarantine useless as a protection, but that it was actually harmful in that it engendered a false feeling of security, and led to the neglect of those sanitary measures which alone could be trusted as a defence against epidemic disease.

In regard to the carrying out of the English system of medical inspection, it was strongly urged that ships should be dealt with as houses are, and that when the infected persons have been removed from them and the vessels disinfected, they should be set free without further detention or isolation. Again, in regard to the so-called medical inspection, it was urged that the medical officer should examine every case of sickness on board vessels from foreign ports, and not merely those on board vessels on which plague, cholera, or yellow fever were suspected to exist, the suspicion being derived from the report of the captain or of a Custom House officer.

Finally, the importance of the sanitary condition of the vessels themselves was insisted on, for the power of a ship to act as a carrier of disease depended greatly upon the arrangements made for maintaining health on board. A foul ship was more to be dreaded than anything in her cargo.

